# Children and Urban Green Infrastructure in the Digital Age: A Systematic Literature Review

**DOI:** 10.3390/ijerph19105906

**Published:** 2022-05-12

**Authors:** Shengchen Yin, Dena Kasraian, Pieter van Wesemael

**Affiliations:** Department of the Built Environment, Eindhoven University of Technology, P.O. Box 513, 5600 MB Eindhoven, The Netherlands; d.kasraian@tue.nl (D.K.); p.j.v.v.wesemael@tue.nl (P.v.W.)

**Keywords:** children, urban green infrastructure, interaction, digital technology, literature review

## Abstract

In the digital age, time spent outdoor in green areas is significantly decreasing for children living in cities. With the advent of digital technology, a series of digital tools are gradually integrated into children’s lives and act as a double-edged sword: on the one hand, an increasing number of children tend to stay at home and play digital games instead of interacting with nature; on the other hand, new digital technology is increasingly being used to engage children with outdoor activities. A host of studies have investigated children’s behaviour in the natural environment. However, a systematic literature review of children’s interaction with the urban green infrastructure (UGI) and the respective role of digital environment, based on a theoretical framework that explicitly takes the multi-level determinants and individual-level mechanism of behaviour change into account does not exist yet. This work provides a conceptual framework that covers various determinants, such as motivation, capability, and opportunity related factors of children’s behaviour in terms of their UGI interaction at the city and neighbourhood levels, while taking into account the individual-level mechanism of behavioural change and the role of the digital environment. The framework is used to systematically review recent international empirical evidence on the determinants of children–UGI interaction. The results are useful for laying the theoretical foundation for future empirical research on children–UGI interaction, specifically in the presence of digital interventions. They also provide urban/digital intervention designers and policymakers with theory-based design and policy guidelines for the creation of child-friendly UGI.

## 1. Introduction

As crucial parts of urban areas, green spaces and networks contribute to the balance between cities and nature, and provide healthy ecological environments and space for various activities, experiences and memories for children [1]. Green spaces are especially beneficial to children’s wellbeing and their personal development.

Green spaces in the city, or more specifically “Urban Green Infrastructure (UGI)” here, refers to an interconnected network within, around and between urban areas, especially the publicly accessible open urban or natural green spaces that are experienced and used by children at the city and neighbourhood levels. Children–UGI interaction can occur through accessibility and exposure to nature (i.e., the existence of specific natural elements and the conditions of direct contact with nature), and engagement with it (i.e., the intentional connection to nature through outdoor activities) [2].

However, for children living in cities, outdoor time is increasingly reducing over the past several decades. One of the possible reasons for this phenomenon is that the quantity and quality of the UGI in most countries have not kept up with expanding cities and the current rate of urbanization [3].

Meanwhile, due to the increasing pervasiveness of digital games, children in cities are likely to spend more time facing electronic screens indoors instead of interacting with nature. This unhealthy lifestyle can cause problems, such as attention deficit and hyperactivity disorder (ADHD), child obesity, stress and cognitive functioning issues [4].

Many studies have investigated children’s interaction with the outdoor environment. A literature review by Aziz and Said (2012) [5] identifies the determinants of children’s use of immediate outdoor environments as demographic, social, and physical factors, and public designs. Meanwhile, new technology is becoming significantly relevant and is increasingly used to stimulate children-nature interaction [4], acting as a facilitator for children–UGI interaction rather than a barrier. However, there is a lack of a behavioural change framework that explicitly considers the role of the digital environment and individual mechanisms of behaviour change, while controlling for the conventional personal, socio-cultural, physical and policy factors. Moreover, few works have empirically investigated the potential positive role of digital interventions in children–UGI interaction. Thus, evidence on the mechanisms through which digital interventions encourage children–UGI interaction, and their outcome for children’s health is limited. This paper’s main research questions based on the above gaps are:

- How can children–UGI interaction be conceptualized while including the potential positive role of the digital interventions?
- What are the determinants of children–UGI interaction, and what is the potential positive role of digital interventions in this interaction according to the state-of-the-art literature?

We answer these questions by (i) suggesting a new conceptual framework that covers various determinants of children–UGI interaction; (ii) using this framework to identify these determinants, including the role of digital environment, through a systematic state-of-the-art literature review. Notably, while acknowledging the potential negative impacts of digital environment on children–UGI interaction, we focus on its potential positive contributions.

The findings are useful for laying the theoretical foundation for future empirical research on children–UGI interaction, specifically in the presence of digital interventions. They also provide urban/digital intervention designers and policymakers with theory-based design and policy guidelines for the creation of child-friendly UGI.

## 2. Theoretical Framework

Behaviour represents the interaction between individuals and their external changes (e.g., social or ecological events), or activities that are functionally mediated or shaped in/by the individuals’ living environments [6]. Children–UGI interaction is a kind of behaviour and influenced by many factors. Various theories describe the determinants of behaviour. Interventions are more likely to be effective if based on determinants identified by theories of behaviour [7]. Here we focus on two relevant and widely applied behaviour models, namely the social ecological and the COM-B model.

### 2.1. The Social-Ecological Model

The social-ecological model is a useful theoretical framework for comprehending the multiple and interacting determinants of behaviour. It contains multiple levels of influence that can be included in the process of health promotion on a spectrum from intrapersonal to policy levels [8]. The intrapersonal level is at the model’s core and is concerned with a person’s capabilities, knowledge, and skills. This level can be influenced by the interpersonal level (i.e., an individual’s relationship with family and friends), the organizational level (which encompass the contact opportunities with people in different organizations like schools and workplaces) and the community level (i.e., the collection of various organizations). Furthermore, the physical environment (including the natural and the built environment) and finally the policy environment (which contains not only formal legal actions taken by local, national, or federal governments, but also informal local policies/rules) are influential. The layers of the social-ecological model can be broadly defined as the personal, socio-cultural (containing the intrapersonal, organizational and community levels), physical and policy levels.

### 2.2. The COM-B Model

The COM-B model is another behaviour theory which aims to capture a wide range of determinants [9]. It comprises three behaviour components: capability, opportunity and motivation. Capability (C) refers to an individual’s attributes, containing physical skills and psychological abilities. Opportunity (O) covers all external chances in an environmental system that together with capability make the behaviour (B) happen. The core of this model is motivation (M), which is a mental process that energizes and directs behaviour and can be influenced by opportunity and capability. Motivation can be automatic or reflective. The first involves the emotions and impulses that arise from associative learning and/or natural personality, and the latter is inspired through evaluations and plans. The relationships between the components of the COM-B model are correlative. Here, capability and opportunity are argued to influence the relationship between motivation and behaviour [10].

The COM-B model does not directly refer to the role of the digital environment in behaviour change. Khalilollahi et al. (in press) [11] contend that the digital environment can provide opportunities for behaviour change in combination with the physical and social environments. They expand the original COM-B model to include the role of digital environment as a new sub-section of opportunity.

### 2.3. A New Conceptual Framework

Both discussed models are useful for identifying the determinants and mechanisms of behaviour change. However, because of their different backgrounds, each framework emphasizes a somewhat different set of determinants and mechanisms. On the one hand, the social-ecological model, which has a background in public health and health geography, puts more emphasis on environmental correlates [12]. It clearly outlines people’s dynamic interaction with the environment, and the potential impact of the environments on individuals’/groups’ wellbeing [13]. Thus, it is widely used in health promotion since the 1980s. Unlike the COM-B model, this model does not address individual-level behaviour components. However, behaviour change interventions are more likely to be effective if they target behaviour components [9]. On the other hand, the COM-B model, which is rooted in behavioural sciences, suggests an individual-level mechanism where behaviour is the result of the interplay among three behaviour components (capability, opportunity, and motivation). So far, this theory has been effectively applied in many recent studies of (children’s) health-related behaviours [14,15]. The COM-B model provides the theory for behaviour components that can be targeted in interventions. However, it is less extensive than the social-ecological model regarding the environmental determinants of behaviour change from macro to micro levels.

We believe these two models can complement each other. Thus, we combine them to explain children–UGI interaction in a more extensive manner that addresses the environmental determinants of behaviour change in relation behaviour components. Several works have used a combination of these two theories before to investigate the determinants and mechanisms of a specific behaviour change [16,17] (van Kasteren et al. 2020; Hunter et al. 2020). However, a combination of these two models has not been applied to explore the mechanisms of children–UGI interaction.

**Figure 1:**
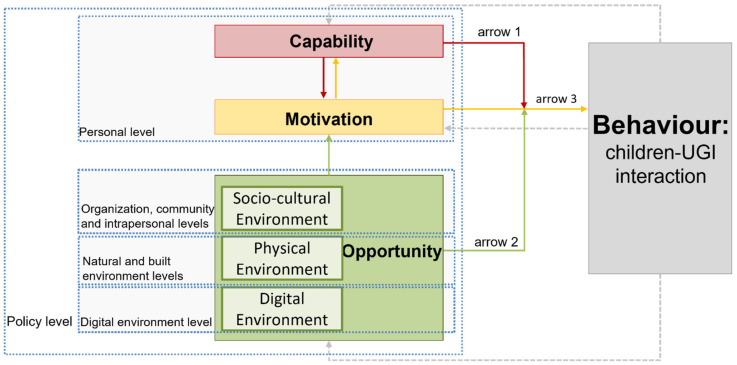
The suggested conceptual model of children–UGI interaction determinants that brings together the COM-B model [10] and the social-ecological model [8] while including the role of digital environment [11].

Thus, we suggest a new theoretical framework that is achieved by mapping the layers of the social-ecological model onto the COM-B components of the Behaviour Change Wheel (BCW) (see Figure 1) to explore the determinants and mechanisms of children–UGI interaction. Based on West and Michie (2020) [10], motivation has a direct influence on behaviour (arrow 3 in Figure 1), while capability and opportunity can affect the relationship between motivation and behaviour (arrows 1 and 2), rather than behaviour itself. Furthermore, the greater the capability and opportunity, the more likely a behaviour is to occur. Here, capability and motivation correspond to the individual level while opportunities are linked to the socio-cultural (containing the intra-personal, organizational and community levels of the social-ecological model), physical, and digital environment levels.

Digital interventions can influence the relationship between children and their behaviour in their living surroundings [18]. This can happen through “digital behaviour change interventions (DBCI)”, which apply digital technology to encourage behaviour change [19]. There are various technologies that can be used to support behaviour change, and they can be classified in different ways. Depending on the type of UGI interaction, the digital interventions can be classified into: digital immersive experience (e.g., holographic technology, virtual reality), and augmented reality interaction (e.g., small pre-set devices, self-portable mobile devices with apps) [20]. Based on the extended COM-B model, the digital environment enables digital interventions and can provide opportunities for behaviour change in combination with the physical and social environments [11].

Attention should be paid to the role of policy. Policy can restrict and indirectly affect the behaviour [21]. It is not directly related to the individual’s participation in the environment, but it influences the events occurring in one or more environments [22]. We include the role of policy as an exogenous factor at the highest level that can potentially influence all lower levels. This is in line with Michie’s categorization of policy in the outermost circle in the BCW and Bronfenbrenner’s inclusion of policy in the “exosystem” which is the larger system of influential environment factors encompassing the micro and meso-systems (see Michie, 2011 [9] and Bronfenbrenner, 1979 [23]).

## 3. Methodology

This literature review follows the PRISMA guidelines and includes empirical studies from January 2005 to March 2021. Our inclusion criteria focus on papers that investigate:

(1) Healthy children (5–11-year-old) living in urban areas. During this age band children are often physically active and autonomously explore their living surroundings where they spend large periods of time outside of home and school. Furthermore, the relationship between children and nature is argued to be most positively impacted before the age of 11 [24].
(2) One or several of the following: motivation-, capability-, opportunity-related determinants of children–UGI interaction; the potential positive roles of digital interventions and the role of policy in this interaction; the outcome of UGI interaction for children’s wellbeing in terms of physical, social, mental wellbeing and cognitive development.

Search strings were based on a combination of the following keywords:

- The UGI-related keywords (“urban green infrastructure” OR “built environment” OR “(neighbourhood) park” OR “natural playground*” OR outdoor OR nature* OR “green space”).
- Target group-related keywords (child* OR kid* OR “primary school student*” OR pupil*).
- Intervention-related keywords (digital OR technology* OR mobile OR game* OR smart OR “physical/social environment”). Here, the asterisk (*) acts as a truncation symbol to denote the derivatives of the word.

**Figure 2:**
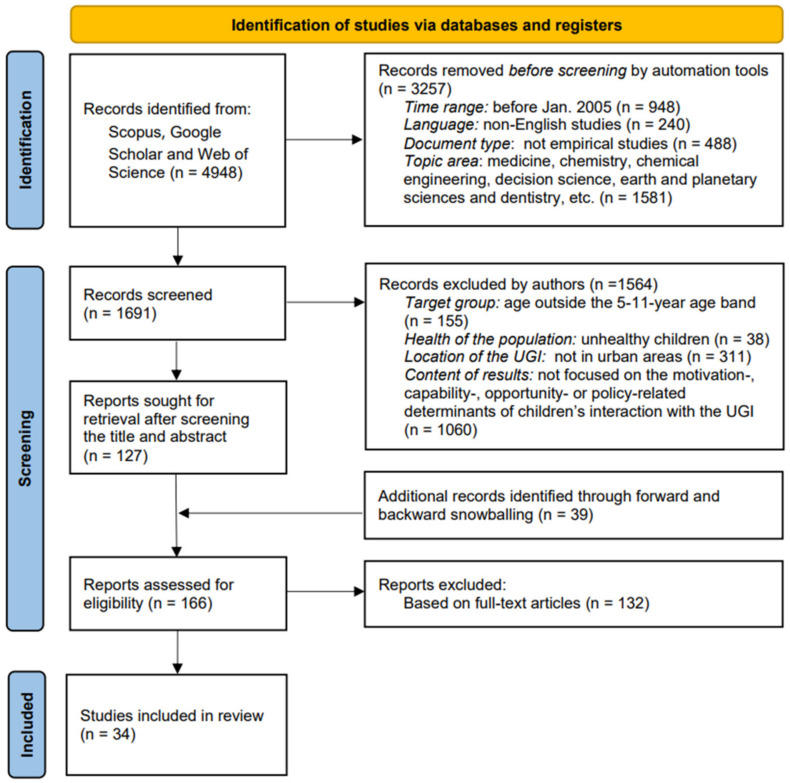
The flow diagram of the study selection process based on the inclusion criteria and the PRISMA guidelines.

Figure 2 demonstrates the study selection process based on the inclusion criteria. The systematic literature search in Google Scholar, Web of Science and Scopus resulted in 1691 studies that met the requirements of time range, language, data type and topic areas. Subsequently, 127 studies were identified based on title and abstract screening. An additional 39 records were found via snowballing. Finally, after full-text reading, 34 results were chosen that met the eligibility criteria of the systematic literature search.

## 4. Results

### 4.1. Study Characteristics

Table A1 provides a detailed overview of the reviewed studies’ characteristics. These studies concentrate on the environments where the children’s growth occurs, from their living neighbourhood to their schools and the natural environments in urban areas they get exposed to.

According to Michie (2011) [9], interventions can change behaviour through one or several “functions”. The reviewed papers show that digital interventions primarily encourage children–UGI interaction through education, persuasion, environmental restructuring and enablement functions. The potential positive functions of digital interventions according to the reviewed papers are shown in Table A1 and discussed below in detail.

### 4.2. Findings

The determinants identified by the reviewed papers were categorized according to the suggested conceptual framework under three behaviour components: motivation, capability and opportunity. The categorization was based on the following: if the determinant was related to children’s physical and psychological abilities to interact with the UGI, it was mapped onto the capability component; if the determinant was concerned with the individual drive-related attributes that directly influence children–UGI interaction, they were included in the motivation component; if the determinant was relevant to external opportunities for socio-cultural/physical environment support, it was mapped onto the opportunity component.

Each section starts with the empirical findings on the component-related determinants of children–UGI interaction identified by the literature and then continues with the potential positive functions and examples of digital interventions encouraging children–UGI interaction in relation to the discussed COM-B model component. All influential determinants as identified by the literature are included in order to not overlook any potential correlates. Overall, 14 determinants were identified from a total of 34 studies. Table A2 provides an overview of the determinants and their frequency of appearance in the literature. It also shows the association of each determinant (positive/negative/not significant) with children–UGI interaction. Accordingly, it can be seen whether an identified determinant, based on the reviewed literature, can be advantageous for children–UGI interaction or not.

### 4.3. Motivation and Children–UGI Interaction

#### 4.3.1. Automatic Motivation: Children’s Interests and Desires at Different Ages

Children’s motivation is the main driver of their interaction with the UGI. Motivation is a result of the interplay between capability and opportunity. When attempting to change behaviour by changing motivations, the key target is the momentary wants and needs which would be experienced at the moment when a specific behaviour takes place [10]. **Children’s interests and desires** represent instinctive, drive-related and effective processes and are crucial to motivating children–UGI interaction. Interestingly, kids of different ages have diverse motivations to engage with natural activities [25]. In particular, young children (5–8 aged) are intrinsically more enthusiastic and motivated by opportunities for exploration, imaginary role-play, and creative/adventurous activities on a small scale, while among more mature children (8–11 aged), there is more tendency to activity, mobility and competition in bigger teams like doing various sports activities together [26,27]. Moreover, peer interactions play a significant role in younger kids’ automatic motivation. A study shows that the energetic and playful interactions are most often observed among younger children in pair and group interactions [28].

#### 4.3.2. Reflective Motivation: Children’s Emotional Evaluations

Children’s reflective motivation includes their **emotional and self-conscious processes**, like evaluations and plans to motivate them to the UGI. Specifically, it refers to whether children regard their decision making to interact with the UGI as right or wrong, good or bad, beneficial or harmful. For example, children express strong preferences and evaluations for the UGI’s natural elements (like shading trees and beautiful flowers). On the contrary, Loebach and Gilliland (2010) [29] report that children’s recognition of poor aesthetics or conditions, such as broken down or unkempt places, challenges their motivation and their resilient sense of ownership regarding their surroundings.

#### 4.3.3. The Potential Functions of Digital Interventions for Stimulating Children’s Motivation

Children’s motivation can be achieved through **education** and **persuasion** functions. Education refers to using digital guidance to increase children’s motivation to engage in environmental education activities and raise their environmental knowledge. Studies show that digital technology has gradually moved from classrooms to the UGI to which children are exposed, and has been widely used in outdoor environmental education [18,30]. New technologies could foster children’s unique outdoor learning experiences, leading to positive educational influences [31]. An example is a smartphone application that uses geocaching to create outdoor learning experiences related to science, technology, engineering, and math (STEM) [32]. Through adventures, players explore and learn about nature by discovering caches located near plants that relay stories about STEM concepts. Another example uses portable digital tools connected through a wireless network that enable recording/collecting natural data and observations about children’s surroundings. This encourages them to form a deeper association of environmental outdoor education and raise their environmental literacy [33]. Children’s interaction with the natural world can also be increased by exploring and learning about animals, as in the case of e-Trailguide, which is an electronic book served as a self-guiding device [30]. This tool uses embedded prompts and activities throughout the whole trail as strategies to cultivate children–UGI interaction.

Digital interventions can positively influence children’s motivation for UGI interaction. The persuasive function of digital interventions can take place through communication strategies like using imagery to influence children’s feelings and attitudes or stimulate their motivation. In an interesting case, the researchers develop a new smart device, GAIA, which is installed on top of outdoor objects, such as trees or streetlights. When touched, it tells nature-related stories to guide children in a treasure hunt for natural elements [34]. The researchers also present a collaborative card-based game to involve children as co-designers of a smart natural ecosystem and motivate them to spend more time at natural activities. ABBOT is another digital interactive device that enables kids to take pictures of what they have found during their environmental explorations, thus motivating them to explore nature [35]. Finally, McClain and Zimmerman (2016) [30] report that an iPad-based e-Trailguide can promote learners’ engagement with outdoor activities through the incorporation of textual prompts and relevant questions.

### 4.4. Capability-Related Determinants of Children–UGI Interaction

#### 4.4.1. Physical and Psychological Capability: Children’s Independent Mobility and Their Perception/Memory of and Familiarity with Nature

Capability-related factors influence children’s motivation to interact with the UGI. **Children’s independent mobility** shows a positive relationship with their connection to the UGI [36,37]. Here, children’s independent mobility means that they have the physical and psychological ability and the permission to move around and perform activities freely and independently without any supervision.

Notably, parental concerns on children’s safety can influence their autonomous mobility. In other words, next to children’s personal physical and psychological capability, the perceptions of their caregivers can restrict children–UGI interaction. Thus, children’s independent mobility can be considered as both a capability-related factor as well an opportunity-related one at the socio-cultural environment level.

For both boys and girls, higher levels of independent mobility are related to higher participation in physical activity contexts [36]. Furthermore, children’s independent mobility levels vary with their age and gender. Children have limited freedom and their access to the UGI is restricted [38]. Additionally, it shows that boys have higher independent mobility and visit neighbourhood, mini- and community parks more often than girls [39]. These differences are mainly due to the parents’ perception of their children’s personal health when children are engaging in outside activities, which greatly affects their possibilities and desires to approach the UGI.

The children’s psychological capability to connect with nature varies between age groups and is related to children’s cognitive development. Children’s mental functioning and psychological capability are reflected in their **perception/memory of, and familiarity with the UGI**, and significantly influence their interaction with UGI. Studies have found that children’s perception of the contact with nature and their nature experience—consisting of enjoyment of nature, empathy for creatures, sense of oneness and responsibility—positively promote their strong interests in performing active behaviours in the UGI, and ultimately enhance children’s physical and psychological health [25,29]. According to Tugurian (2015) [40], children’s experience and emotional feedbacks of their living natural environment are reflected in their feelings of freedom and comfort in natural zones and lead to children’s stress relief and mental wellbeing improvement. Additionally, children tend to visit the same UGI areas repeatedly due to the presence of psychological affections. Thus, the familiar surroundings that children experience may result in them having more comfortable feelings in these natural areas [38].

#### 4.4.2. The Potential Functions of Digital Interventions for Stimulating Children’s Capability

Physical and psychological capability can be achieved by the **enablement** function [9]. Digital interventions can add possibilities or reduce obstacles with the aim of increasing capability/opportunity for children–UGI interaction. This can be in form of outdoor or learning-from-nature activities. For instance, mobile applications like Agents of Nature can engage children with activities in local parks and increase their independent connection with nature [26]. In another example, a digital mind map helped children increase their engagement and participation during outdoor learning experiences [31]. Finally, Huang et al. (2010) [41] observed that the use of mobile planting learning system can enable children to increase their knowledge about plants by participating in assigned learning activities in the field.

### 4.5. Opportunity-Related Determinants of Children–UGI Interaction

#### 4.5.1. The Socio-Cultural Environment

Children living in urban areas increasingly have less access to nature, and the experience and movement (range) of quite a lot of children is limited or even restricted. Not only **households’ anxiety about children’s safety** [39,42], but also **schools’ and teachers’ management of children** could reduce their available time for UGI interaction. This is evident in children’s remarks on how rarely they have the opportunity to contact with the natural world except for during school recess [40]. However, **parental presence, company and engagement** can increase children’s immersion in and enjoyment of nature activities. Parents are significant “enablers” of child–nature interactions and can significantly motivate their kids to connect with the natural world [27]. Chen (2020) [43] argues that parents’ presence and company as an emotional support is much needed by children when doing outdoor activities. The UGI can provide an intergenerational space for both children and their parents to share moments of happiness and retain family memories together. It is claimed that children enjoy doing family-oriented activities in urban natural areas [27].

Additionally, **children’s social and cultural background** has a fundamental influence on how they perceive, experience and use the UGI [44]. Children of different genders, races, ethnicities and residence locations have different UGI preferences [39]. In particular, gender could influence the relationship between social attributes and urban form characteristics of the surrounding neighbourhood and the UGI use [45]. Similarly, children’s socio-cultural background could also be decisive. This can be proxied by their residential location. A study in three different areas of Sweden indicates that different levels of social development in various geographical surroundings could possibly stimulate or hinder children’s participation in child-friendly environments [44]. The findings indicate children living in the metropolitan areas of Sweden value safety more, while children from the country’s less urban north pay more attention to the urban and environmental qualities.

Additionally, children’s **social networks** of their friends, peers or other children to play with, are related to an increased likelihood of opportunities for them to do more cooperative outdoor activities, and enhance their play performance and problem solving skills in the UGI [27,28,37]. Chen et al. (2020) [43] report that many UGI areas are being created more exclusively for kids to extend their peer and friendship networks. They find that the presence of other children prolongs outdoor time and prompts children to make plans to complete specific tasks and play together more often. Another example shows that collective playing activities like building a tree house with friends results in a strong sense of accomplishment for children [27]. Such community-oriented social networks provide children with more opportunities and desires to interact with the UGI.

#### 4.5.2. The Physical Environment

The physical availability of the UGI is the first condition for children–UGI interaction [46]. Based on the reviewed literature, the physical (built and natural) environment characteristics can be categorized into four groups. Firstly, **the natural features of the UGI**—such as the presence and amount of vegetation, water and sand—are the main and favourite elements of the UGI for children’s play [2,47,48,49]. In particular, water-related settings are the most highly interactive areas enjoyed by children, mainly because of their various sounds, changes in state, special feelings of wetness and different functional uses like splashing, floating objects and pouring [45,50]. Another popular play material in the UGI is sand, which provides more intriguing opportunities for children to dig, sculpt and draw, improving their imagination and creativity [28,50]. Interestingly, potentials for tactile interactions with the UGI’s natural elements can also provide a physical environment opportunity. It is shown that children have a clearer cognition of their surroundings when touching the natural things physically, such as picking up leaves, touching branches or the surface of the shell [28]. These natural components of the UGI can support kids’ spontaneous outdoor activities and further influence their wellbeing and integral development [51]. Additionally, the morphological diversity of UGI surfaces increases the variety of spatial conditions, leading to more opportunities for children’s exploration and nature experience, whereas uniformity and regularity of surfaces could reduce these opportunities [51]. For instance, it is shown that the diversity of topography with slopes and roughness is more valued by children aged 8 to 11 years old for playing and resting [50].

Additionally, the second and third characteristics of the UGI physical environment appear to be **the UGI’s accessibility and size**, respectively. In particular, proximity to the UGI is positively associated with physical activities and children’s general health [25,38,39,49,52]. Similarly, larger UGIs are more likely to attract kids. Thus, an increase in park size and the neighbourhood’s UGI proportion is linked to higher use by children [45,53].

The fourth category involves **the characteristics of play equipment and recreation facilities** present in the physical environment. If the playing equipment are designed to be adventurous and challenging (some to be used with a certain guidance level), they can become one of children’s favourite elements in the UGI, attracting them to free play or exploration [2,39,47,48]. A study shows that the majority of kids mostly interact with the designed and organized UGI (such as playgrounds, sports fields, schoolyards and parks) rather than undesigned and disorganized places like streets and sidewalks [54]. Loukaitou-Sideris and Sideris (2010) [39] find that park equipment is used more frequently by girls than boys due to significant gender, racial and ethnic differences in preferences for playground equipment in the UGI. Formal and informal sport-related equipment are more popular among boys, whereas girls prefer non-sport related facilities and play equipment, such as swings, monkey bars and water features [55].

In addition, **places for nature-related activities in/around schools** can facilitate children–UGI interaction. Children can not only join the outdoor classes taught by teachers to understand the diversity of natural features and local environmental issues [56], but also do after-school activities, including catching and observing insects, following animal tracks in mud and snow, and observing the annual cycle of diverse pond wildlife [40].

#### 4.5.3. The Potential Functions of Digital Interventions for Stimulating Children’s Opportunity

Opportunity can be provided for children–UGI interaction by the function of **environmental restructuring**. Digital interventions can have an “environmental restructuring” function, where digital technology is used to change the physical and social contexts to motivate children–UGI interaction. It has been shown that personal digital assistants (PDAs) combined with wireless communication technology can stimulate children to enthusiastically engage in assigned learning activities. Furthermore, they provide opportunities for children to observe and absorb peers’ reflections and perceptions in order to improve their social interactions and environment cognition [41]. For instance, Nature Collections, a mobile app, has been successfully applied in promoting close observations of the natural elements in children’s living surroundings, and has also prompted children’s nature-related conversations with their peers [28]. Loebach and Gilliland (2010) [29] use an online software to improve children’s accessibility to, usability of and safety in the urban environment. They identify children’s preferred “spot” for creating a physical soundscape by their GPS-defined locations and mark these places in the surroundings. Using authoring software on handhelds and an editing interface on networked PCs, children can be motivated to create satisfying soundscapes when accessing their preferred “spot” in the UGI.

### 4.6. The Role of Policy in Children–UGI Interaction

According to the BCW framework, the role of policy is to enable or support the implementation of specific interventions [9]. Policy can take different forms, including environmental/social planning and regulations (see Michie, 2011 for more elaboration on different policy types). Local and (inter)national policies are significant for the further development and implementation of child-focused environmental planning [57].

Several studies have mentioned initiatives that could potentially contribute to children–UGI interaction. For example, the City of London has recently adopted a Children’s Agenda and has established a Child Network to identify the most urgent needs of children in the local neighbourhoods and address them through policy and environmental changes [29]. Similarly, Auckland City has introduced a children-first approach that encourages policies that consider children’s needs [58]. Another example is the “Opportunities for Children in Urban Spaces (OCUS)” audit protocol, which emphasizes children’s rights and interests regarding the UGI by enabling their independent mobility [51]. This protocol can also evaluate children’s social, emotional and functional affordance, and also the circumstances of access to the UGI.

However, the contribution of policy to children–UGI interaction has hardly been investigated in empirical literature. There is a need for empirical investigations of the influence and success of various policy types regarding children–UGI interaction to guide evidence-based policy making.

## 5. Conclusions, Policy and Design Implications, and Avenues for Future Research

### 5.1. Conclusions and Policy and Design Implications

**Figure 3:**
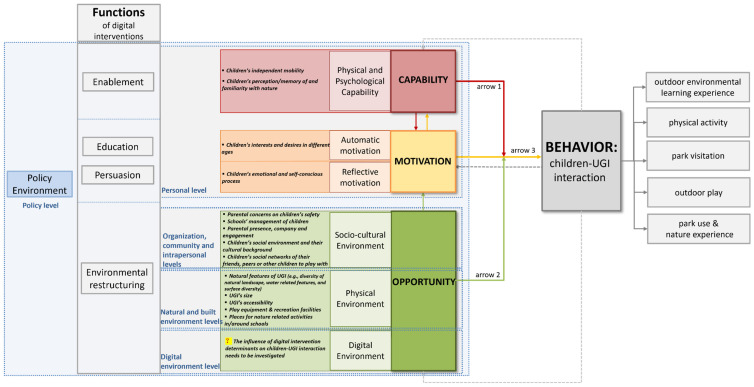
The conceptual model of the determinants of children–UGI interaction and the potential functions of digital interventions.

This systematic review of the state-of-the-art international literature reviews the motivation-, capability- and opportunity-related determinants of children–UGI interaction. Furthermore, it identifies the potential positive role of the digital environment in this interaction both conceptually and empirically. Its specific contribution to the research field is three-fold. Firstly, it creates a new conceptual framework based on two existing behaviour models, i.e., the social-ecological and COM-B models, that can be applied to children–UGI interaction in the presence of the digital interventions. This adds to the theory of behaviour change and is beneficial for public health and health geography researchers as well as policymakers aiming to promote healthy behaviour. Secondly, based on the recent empirical studies, this paper identifies the crucial factors that influence children–UGI interaction and improve their wellbeing in the digital era. It presents these factors in a multi-level structure consisting of child-related (personal) factors, as well as external environments (physical, socio-cultural, digital, and policy environments), in relation to COM-B model components (Figure 3). Third, it identifies the functions of digital interventions (i.e., enablement, education, persuasion, and environmental restructuring) that can target various behaviour components and eventually increase children–UGI interaction.

The above contributions can benefit the urban/digital intervention designers, planners, and policy makers in developing effective interventions based on the identified correlates of children–UGI interaction and functions of digital interventions linked to behaviour change components. For instance, our work shows that children–UGI interaction can be improved by increasing children’s physical and psychological capability. To do so, designs can consider children’s independent mobility and their perception of nature and familiarity with it. Furthermore, as we demonstrate, children’s capabilities can be enhanced by the enablement function of digital interventions that add further possibilities or reduce existing obstacles. This could be done through the use of self-portable mobile devices with apps that stimulate children to engage in UGI-related recreational and learning activities and enable their independent connection with nature.

The results of this review show that the majority of the selected studies focus on the influence of physical/socio-cultural environments and propose several approaches to measure and/or improve this interaction. The following section outlines the recommendations for designing and planning the UGI in order to stimulate children–UGI interaction, under COM-B model components.

We start with recommendations for **capability- and motivation-related** determinants. First, children’s independent mobility is a significant factor that could be supported by initiatives that enhance parents’ perception and the physical setting where children–UGI interaction occurs. These are further discussed under the suggestions for socio-cultural and physical environments below. Furthermore, enhancing children’s affective attitudes and interests toward nature are important and beneficial to their long-term development. This can be done by providing opportunities such as environmental education and nature-based activities, and shaping family values towards nature [25]. Additionally, designs should meet the need of children with different physical and psychological capabilities e.g., in terms of the material barriers like steps, kerbs, step gradients, uneven surfaces [51].

**Opportunity-related** recommendations address three areas. First, *suggestions for the socio-cultural environment*: Parental experiences and needs should be considered in park design [43]. On the other hand, studies indicate that imposing overprotective parental practices serve and excessive restrictions on children’s outdoor activities stifle their growth [59,60]. Thus, children’s safety initiatives should be balanced with opportunities for child development through risky outdoor activities [61]. Strategies like keeping children “as safe as necessary” instead of “as safe as possible” [62] have a potential value for child development while also ensuring their safety. Additionally, the presence of children’s peers and friends is critical for encouraging UGI contact. Thus, providing opportunities for cooperative activities in the UGI like building a tree house with friends are recommended. There is a need for the adoption of policies and regulations to increase the use of the UGI based on gender-related factors since gender seems to be an important element and moderator in children–UGI interaction.

Second, *suggestions for the physical environment*: Figure 3 shows the factors that encourage children–UGI interaction. Offering various activities and facilities in parks is a minimum requirement for planning children’s physical environment [39]. Urban designers and planners are recommended to shift their thinking away from manicured park lawns and high-quality manufactured play equipment toward restructuring the local natural environment for children where children can come to contact with small animals like insects and local plants [63,64].

The link between park use and urban form characteristics can be translated into policies and design guidelines to plan the outdoor environment for enhancing children–UGI interaction [45]. More specifically, proximity and accessibility to the UGI are important correlates of children–UGI interaction [45,49]. Thus, UGI should be designed to be as accessible as possible to children by considering the surrounding neighbourhoods, and the pedestrian and cycling environments leading to the UGI (e.g., bike lanes, wide sidewalks, overpasses, traffic lights, residential culs-de-sacs, etc.) [39]. Examples are child-friendly walkable and visible pathways through the neighbourhood connecting home/school to the UGI that could be planned with cultural/natural element guides and signs [65]. Child-friendly signs with suitable height and content could show the routes and make the trip a learning and fun experience. Additionally, to motivate children’s adventurous play, the design and quality considerations of the designed areas in the UGI should be combined with dramatic light, shadows, natural climbing and rocking elements, to make it more mysterious for children’s further exploration [47]. Children of different ages and gender vary widely in using UGI facilities [45] UGI designers should account for these differences rather than seeing children as a homogeneous group with uniform needs, supposedly satisfied by the provision of standardized facilities [66]. Particularly, the UGI equipment should not only include (in)formal sport-related features for boys but also non-sport features designed for girls [55]. Besides, planners should design age-appropriate facilities that meet safety requirements [37]. From parents’ perspective, adding urban furniture around the neighbourhood for parents and designing more family-oriented activities could facilitate observing children when playing [39,54]. Regarding schools, it is recommended that they are designed to maximise children’s connection to plants and animals [56].

Third, *suggestions for the digital environment*: Findings from the 12 studies on the potential roles of digital interventions in stimulating children–UGI interaction show that digital interventions (such as digital immersive experiences and augmented reality interactions) can facilitate children’s interaction with the UGI through several functions. The education and persuasion functions of digital interventions can stimulate children’s motivation. Their enablement and environmental restructuring functions can enhance children’s capabilities and opportunities, respectively. Furthermore, technology can play a significant role in assisting parents in enabling their children’s interaction with nature [27]. Some studies recommend teachers and educators to integrate digital technology into programs as educational instruments [18]. Thus, children’s scientific research skills and experiences in the natural world could be enriched by encouraging them to use technology outside [33].

### 5.2. Avenues for Future Research

This review reveals several limitations in the existing literature, and consequently corresponding avenues for future research. Firstly, recent studies on children–UGI interaction increasingly focus on the role and application of digital technology. This paper conceptually frames this potentially positive emerging roles and supports it with the findings of twelve empirical studies. However, the impact of digital interventions such as digital immersive experience and augmented reality interaction designed to promote children–UGI interaction is under-researched. Therefore, further empirical case studies are needed to explore the determinants of digital environments that influence children–UGI interaction and the potential impact of various technology types on it.

Secondly, most studies investigate the outcome of socio-cultural and physical environments’ opportunities on children’s physical health. However, the contribution of these determinants to children’s mental health and cognitive development still requires further investigation.

Thirdly, the majority of the reviewed papers are carried out at the neighbourhood-level separate green areas in cities. However, large scale city-level UGI like a city’s park system could contribute differently to children–UGI interaction and their health. Thus, future research could be extended to the city region and investigate the role of different determinants of children’s interaction with city-level UGI and its implications for their health.

Finally, while some recommendations for policy makers and urban planners exist, empirical findings on the impact of various policies on children–UGI interaction are very scarce. In other words, studies mostly focus on children’s individual and external environment aspects which are more closely related to them, rather than the potential contributions of policy. This is mainly due to the fact that policy contributions usually appear with a delay in time and the outcomes could be influenced by a host of other external factors that are hard to control for (e.g., the socio-cultural context). Thus, further extensive empirical research is needed regarding the role of the policy environment in improving children–UGI interaction. The findings can contribute to the much more needed evidence-based policy formulation that encourages children–UGI interaction in the digital age.

## Data Availability

Not applicable.

## Appendix A

**Table A1:** Overview of the selected studies and their characteristics.

**Study Type**	Peer-reviewed journal publications (29); Conference papers (4); PhD dissertation (1)
**Location**	US (13); Australia (4); Europe (17)
**UGI Level**	Neighbourhood level * (29); City level (5)
**Intervention Type**	Personal capability (2); Social-cultural environment (9); Physical built environment (11); Digital interventions (12); Policy environment (4)
**Research Design**	Qualitative (19); Quantitative (10); Both (6)
**Wellbeing**	Physical (15); Social (8); Mental (5); Cognitive development (8)
**Interaction Type**	Outdoor environmental learning experience (10); Physical activity (9); Park visitation (2); Outdoor play (4); Park use and nature experience (4); Other kinds of behaviour (5)
**Digital Intervention Function**	Environmental restructuring (2); Enablement (11); Persuasion (2); Education (7)

*Notes:* (n) is the number of papers; * includes neighbourhood, schoolgrounds and outdoor gardens.

**Table A2:** The identified correlates and their association with children–UGI interaction.

COM-B	Components	Determinants	Share of Studies Investigating the Correlate (n/N)	Association with Children–UGI Interaction			Share of Studies That Find Significant Association (n/N)
				+	0	−	
Capability	Physical and psychological Capability	Independent mobility	3/34	[36,37]		[38]	3/3
		Perception/memory of and familiarity with nature	4/34	[25,29,38,40]			4/4
Motivation	Automatic Motivation	Interests and desires in different ages	4/34	[26,27,28]	[25]		3/4
	Reflective Motivation	Emotional and self-conscious processes	1/34	[29]			1/1
Opportunity	Socio-cultural Environment	Parental concerns on children’s safety	1/34			[39]	1/1
		Schools’ management of children	1/34			[40]	1/1
		Parental presence, company and engagement	3/34	[27,39,43]			3/3
		Social environment and their cultural background	3/34	[44]	[39,45]	[44]	1/3
		Social networks of their friends, peers or other children to play with	4/34	[27,28,37,43]			4/4
	Physical Environment	Natural features of UGI	8/34	[28,45,49,50,51]	[2,47,48]		5/8
		UGI’s accessibility	5/34	[25,38,39,49,52]			5/5
		UGI’s size	1/34	[45]			1/2
		Play equipment and recreation facilities	6/34	[2,39,47,48]	[54,55]		4/6
		Places for nature related activities in/around schools	2/34	[50]	[40]		1/2

*Notes:* +, positive relation; 0, no relation; −, negative relation.
